# The efficacy of a combination of naproxen and fexofenadine (SJP − 002) to inhibit the symptoms that are associated with viral upper respiratory tract infection: Four case reports of individuals with common cold

**DOI:** 10.1002/ccr3.7682

**Published:** 2023-07-20

**Authors:** Emina Išerić, Thomas A. Dahl, Andrew Scholey, Jacqueline M. Iversen, Joris C. Verster

**Affiliations:** ^1^ Division of Pharmacology, Utrecht Institute for Pharmaceutical Sciences (UIPS) Utrecht University Utrecht The Netherlands; ^2^ Sen‐Jam Pharmaceutical Huntington New York USA; ^3^ Nutrition Dietetics and Food, School of Clinical Sciences Monash University Melbourne Victoria Australia; ^4^ Centre for Human Psychopharmacology Swinburne University Melbourne Victoria Australia

**Keywords:** common cold, fexofenadine, naproxen, rhinovirus, SJP − 002, treatment, upper respiratory tract infection

## Abstract

**Key Clinical Message:**

There is no effective treatment that reduces both the severity and duration of a common cold episode. SJP − 002 (naproxen and fexofenadine) reduced symptom severity by two‐third, and the duration of the common cold episode approximately by half.

**Abstract:**

The common cold is one of the most frequently experienced immune‐related complaints. At present, available treatments have limited efficacy in inhibiting symptoms associated with upper respiratory tract infection, nor do they significantly shorten the duration of common cold episodes. Four case reports are presented of individuals with a common cold. Three of them self‐administered the combination of naproxen and fexofenadine (SJP − 002). Results of one individual were compared to her spouse, who did not take SJP − 002 to treat common cold, while two other individuals took SJP − 002 and compared symptom severity with another common cold episode they experienced previously without taking SJP − 002. SJP − 002 reduced the severity of symptoms associated with upper respiratory tract infection by two‐third and reduced the duration of the common cold episode approximately by half. In conclusion, SJP − 002 reduced the severity and duration of common cold episodes. These findings warrant further investigation of SJP − 002 in double‐blind, placebo‐controlled clinical trials.

## 1 INTRODUCTION

The common cold, that is, upper respiratory tract infection, is an infectious disease characterized by symptoms such as sore throat, runny nose, coughing, sneezing, headaches, and body aches.^1^, ^2^ Although common colds are self‐limiting, people with reduced immune fitness, or pulmonary conditions (e.g., asthma or bronchitis) may develop longer lasting episodes of common cold that may develop into more serious illness, such as pneumonia.^1^, ^2^ Up to 80% of common colds are caused by a rhinovirus.^3^, ^4^, ^5^ Other viruses that may cause common cold include the adenovirus, parainfluenza virus, respiratory syncytial virus, enterovirus, and coronavirus.^3^, ^4^, ^5^ The typical duration of a common cold episode is about 10 days. After infection, individuals follow a typical course characterized by (a) the incubation phase (an average of 1 or 2 days, of which they are usually unaware), followed by (b) the first appearance of symptoms (Days 1–3), (c) the progression of symptoms to peak severity (Days 4–7), and concluded by (d) the remission phase (Days 8–10) when symptoms gradually disappear.^6^ The durations of these phases are variable, and it may take up to 3 weeks for symptoms to disappear and individuals to fully recover and function at full capacity.^2^ Yearly, adults experience an average of 2–3 common cold periods, and children become infected even more frequently.^7^, ^8^ The Global Burden of Diseases, Injuries, and Risk Factors Study, including data from 204 countries, estimated the global number of upper respiratory tract infections in 2019 at 17.2 billion cases.^9^ Overall, the common cold accounted for nearly half of all cases of burden of disease.^9^

The common cold poses a significant impact on the healthcare system (e.g., physician visits and medications)^10^; the symptoms of common cold can also have a significant negative impact on daily activities, mood, and quality of life.^11^, ^12^ Other studies found that the common cold significantly impaired performance of common daily activities such as driving a car.^13^ The common cold is a primary cause of absenteeism (not going to work) and presenteeism (attending work while sick, with a significant decline in function and productivity as a result).^10^, ^14^ About 20 years ago, a US study estimated the annual costs for the US economy between 25 and 40 billion USD per year, with approximately 70–120 million lost work days.^10^ A literature search did not reveal more recent US data. However, a 2010 Swedish study estimated the combined costs of absenteeism (44%), presenteeism (37%), and care giver absenteeism (19%) for the Swedish economy at 2.7 billion euro.^15^ Thus, developing an effective and safe treatment for the common cold could have significant health and economic benefits. Ideally, such a treatment would reduce both the severity and duration of the common cold episode.

The primary methods of preventing infection are behavioral precautions such as regular hand washing with soap or alcohol‐based hand sanitizer, not touching the eyes, nose, or mouth with unwashed hands, and avoiding contact with infected individuals.^1^ There is no vaccine against the common cold.^16^

A great number of treatments are available to treat the common cold.^7^ Natural treatments include herbal products, vitamins, and minerals, but convincing evidence for their efficacy is usually lacking.^7^ A 2013 Cochrane review revealed that acetaminophen (paracetamol) alone may help to relieve some common cold symptoms (nasal obstruction and rhinorrhea), but does not improve others (e.g., sore throat, malaise, sneezing, and cough).^17^ A more recent Cochrane review examined the combination products of analgesics, decongestants, and antihistamines for the treatment of common cold.^18^ The review of 30 studies concluded that the methodological quality and reporting of the studies was often poor. Nevertheless, the currently available evidence suggests that the combination treatments may inhibit common cold symptoms to some extent. Whereas the use of combination treatments containing decongestants was associated with experiencing adverse effects such as dry mouth, insomnia, and dizziness, the antihistamine and analgesic combinations did not produce more adverse effects than the placebo groups. In the Cochrane review, four studies investigating the combination of antihistamines and analgesics were included.^19^, ^20^, ^21^, ^22^ Of these, the three in adults examined combination treatments that were not limited to an antihistamine and analgesic drug only. Rather they included a combination of paracetamol and phenylpropanolamine or paracetamol and diphenhydramine^19^; a combination of ibuprofen, chlorpheniramine, and intranasal interferon^20^; a combination of acetaminophen, caffeine, chlorpheniramine, and ascorbic acid.^21^ A fourth study in children aged 6 to 24 months examined the combination of buphenine, aminophenazone, and diphenylpyraline.^22^ Thus, to the best of our knowledge, no studies have been conducted to evaluate the efficacy of a treatment comprising the combination of a non‐steroid anti‐inflammatory drug (NSAID) and an antihistamine drug (i.e., an H_1_‐receptor antagonist).

SJP − 002, a combination of an NSAID and an antihistamine drug (naproxen and fexofenadine), is currently being developed. By its combined anti‐inflammatory action of the NSAID and the mast cell stabilizing effect of the antihistamine drug, it is hypothesized that SJP − 002, taken when symptoms first appear (Days 1–3), would limit the symptom severity and duration of the common cold before the progression into the more severe active phase (Days 4–7). No decongestant is included in this drug combination, and fexofenadine is a non‐sedative H_1_‐receptor antagonist. Also, naproxen and fexofenadine have been available OTC as individual drugs for many years, and showed to have an excellent safety profile. Therefore, no significant adverse effects are expected from the combination of the two drugs (SJP − 002). Here, four case reports are presented of subjects who self‐administered the combination of the NSAID, naproxen and the antihistamine drug fexofenadine (referred to as SJP − 002 in this article) to treat the common cold.

## 2 METHODS

An individual (Subject 1) reviewed the Sen‐Jam Pharmaceutical website on the Internet and consulted the company about the availability of SJP − 002 for the treatment of common cold. The subject was told that SJP − 002 was not marketed yet, but that naproxen and fexofenadine are over‐the‐counter (OTC) drugs that are freely available in the USA. The subjects decided to buy the drugs at their local pharmacy and self‐administer a single oral dose of 220 mg naproxen sodium, directly followed by a single oral dose of 60 mg fexofenadine HCL, every 12 hours. Although the final dosing should be determined in future clinical trials, these dosages were suggested as these fall within the dose ranges approved by the US Food and Drug Administration (FDA) for OTC use of naproxen sodium (dose range 220 mg‐440 mg) and fexofenadine HCl (60 mg, twice daily). The spouse of the individual (Subject 2) had also experienced common cold symptoms but did not use SJP − 002. Both subjects were asked to report their common cold symptoms to the corresponding author. Two other individuals (Subject 3 and Subject 4) also contacted Sen‐Jam Pharmaceutical after searching the Internet for a treatment of their common cold complaints. They previously experienced a common cold occasion on which they had used no treatments to inhibit common cold symptoms. For the current common cold occasion, they decided to self‐administer SJP − 002. No ethics approval was needed for self‐administration of these OTC drugs. Participants agreed to report their common cold symptoms to the corresponding author and gave written consent to publish their data. For both occasions, the experienced common cold symptoms were reported via phone to the corresponding author. Common cold symptoms were assessed with a modified version of the Jackson Symptoms Scale,^23^, ^24^, ^25^ including the symptoms “runny nose”, “nasal congestion”, “sneezing”, “coughing”, “headache”, “fever”, “chills”, “muscle/joint pain”, and “sore throat”. These were rated on an 11‐point scale ranging from 0 (absent) to 10 (extreme). The sum score of all symptoms was computed, ranging from 0 to 90, with higher scores indicating a more severe common cold.

## 3 RESULTS

During the month of April, 2017, Subject 1 contacted Sen‐Jam Pharmaceutical. Subject 1 and her husband (Subject 2), a middle‐aged man, both suffered from common cold symptoms. Subject 1 was seeking an effective product to inhibit their upper respiratory tract symptoms. Subject 2 reported that he had been exposed to the common cold virus while at a conference. Two days after returning home from the conference and for the subsequent 14 days, he experienced symptoms associated with the common cold. On Days 5–14, Subject 2 took several OTC products (i.e., a cough suppressant, a throat lozenge, and a decongestant). On Day 14, Subject 2 saw his primary care physician who diagnosed him with viral upper respiratory infection, but prescribed antibiotics and steroids. On Day 16, Subject 2 reported the magnitude of his common cold symptoms to the corresponding author. The common cold episode of subject 2 was not resolved on Day 16. His wife, Subject 1, believed she contracted the common cold from Subject 2. The first symptoms occurred 4 days after Subject 2 returned home. Subject 1 self‐administered SJP − 002 every 12 h for 8 doses (i.e., 4 days). She reported experiencing cold symptoms for approximately 7 days. On Day 10, she reported her past common cold symptoms. The symptom severities reported by Subjects 1 and 2 are listed in Table 1.

**TABLE 1:** Common cold symptoms reported by Subject 1 and Subject 2.

Symptoms	Subject 1 (SJP − 002)	Subject 2 (no SJP − 002)
Runny nose	4	0
Nasal congestion	3	10
Sneezing	1	0
Coughing	1	10
Headache	1	8
Fever	0	2
Chills	1	2
Muscle/Joint pain	0	8
Sore throat	0	0
Sum score	11	40

*Note*: Common cold symptoms were assessed on a scale ranging from 0 (absent) to 10 (severe).

Two additional subjects (Subject 3 and Subject 4) evaluated both a common cold occasion without SJP − 002 treatment with an occasion with SJP − 002 treatment. Subject 3 was a 22‐year‐old male subject. Subject 4 was a 57‐year‐old female subject. On the day they started experiencing common cold symptoms, both subjects started self‐administering SJP − 002 every 12 hours for 6 doses. They reported common cold symptoms for both the previous common cold occasion (without SJP − 002 treatment) and the current common cold occasion (with SJP − 002 treatment). The results are summarized in Table 2.

**TABLE 2:** Common cold symptoms reported by Subject 3 and Subject 4.

	Subject 3		Subject 4	
Symptoms	No SJP − 002	SJP − 002	No SJP − 002	SJP − 002
Runny nose	1	0	10	2
Nasal congestion	5	3	10	2
Sneezing	3	3	2	2
Coughing	4	2	2	2
Headache	4	2	5	2
Fever	3	0	3	0
Chills	4	0	3	0
Muscle/Joint pain	3	2	10	2
Sore throat	3	1	2	0
Sum score	30	13	47	12

*Note*: Common cold symptoms were assessed with the Jackson Symptoms Scale. Scores ranged from 0 (absent) to 10 (severe).

**FIGURE 1:**
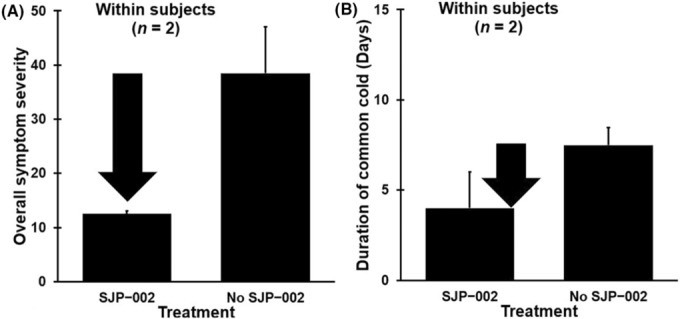
Mean and standard error (SE) of overall common cold severity for common cold occasions with or without treatment with SJP − 002. (A) shows the overall symptom severity; (B) shows the duration of the common cold episode. No significant differences were found.

The average common cold sum scores of Subjects 3 and 4 for the common cold occasion with and without self‐administering SJP − 002 are shown in Figure 1A. A statistical comparison of the two common cold occasions revealed that the sum scores were lower after self‐administering SJP − 002 (a mean [SD] of 12.5 (0.7)) compared to the common cold occasions when SJP − 002 was not taken (a mean [SD] of 38.5 (12.0)). The difference did not reach statistical significance (*p* = 0.212). The reported duration of the common cold symptoms with and without self‐administering SJP − 002 were 5 vs. 7 days for Subject 3 and 3 vs. 8 days for Subject 4, respectively (Figure 1B). The overall shortening in days of experiencing common cold symptoms after SJP − 002 (4.0 days) compared to not using SJP − 002 (7.5 days) did not reach statistical significance (*p* = 0.258).

## 4 DISCUSSION

The case studies presented here suggest that SJP − 002 is an effective treatment that reduces the severity of common cold symptoms. Subjects that received SJP − 002 also experienced a shorter common cold episode, however this difference in duration was not statistically significant. These findings warrant further investigation of SJP − 002 in a double‐blind, placebo‐controlled clinical trial in a larger sample.

After SJP − 002, the overall common cold severity scores were two‐third lower than those without taking SJP − 002. Also, the duration of the common cold period was reduced approximately by half. The implications of these findings, if replicated in a larger trial, are evident. First of all, it would mean the availability of a truly effective medicine that both reduces symptom severity and shortens the duration of the common cold episode. Second, this may result in significant reduction in days of absenteeism and presenteeism. Given the high impact of experiencing common cold on the economy, the latter should not be underestimated. A US study^10^ estimated the annual costs of common cold for absenteeism and presenteeism at 25–40 billion USD. If the duration of the common cold episodes and the severity of experienced symptoms could be reduced by 50%, this would have a profound positive impact on the US economy.

Also from a consumer perspective, there is a clear need for common cold remedies. A 2022 market analysis and forecast by Statista^26^ estimated the global cold and cough remedy market of 2022 at US$39.26 billion, with an expected market increase from 2016 to 2027 of 5.6%. The United States and China (US$10.21 billion and US$9.01 billion, respectively) account for about half of the cold and cough remedy market.^26^

The observed effects of SJP − 002 were in line with expectations. Previous research revealed that the combination of naproxen and fexofenadine was successful in inhibiting side effects of travel and influenza vaccinations (SJP – 003)^27^, ^28^ and reduced the severity of alcohol hangover (SJP – 001).^29^ A likely explanation is the fact that these conditions share several common symptoms (e.g., fatigue, aches and pains, headache). It is thought that the anti‐inflammatory effect of the NSAID is strengthened by the mast cell stabilizing effects of the antihistamine drug. Another NSAID/antihistamine drug combination (SJP – 005, the combination of ibuprofen and ketotifen) showed to be effective in significantly reducing the incidence and duration of discontinuation‐induced morphine withdrawal symptoms in rats, when treatment was initiated 2 days before morphine cessation.^30^ A second study found that rats could withstand higher levels of painful stimuli when SJP‐005 was co‐administered, suggesting a possible opioid sparing effect.^31^ A synergistic effect was demonstrated in a preclinical study demonstrating the effectiveness of another NSAID/antihistamine drug combination (SJP – 002C, the combination of indomethacin and ketotifen) in inhibiting 2019 coronavirus replication.^32^ Together, these studies suggest that NSAID/antihistamine drug combinations could be highly effective in conditions that are characterized by inflammatory responses of the body. For the development of SJP – 002, the combination of naproxen and fexofenadine was selected. Naproxen sodium was chosen because it is available OTC (in contrast to indomethacin and ketotifen, which are prescription drugs in US), due to its safety profile, and twice daily administration schedule. It is believed that naproxen sodium will reduce symptoms of headache, body/muscle aches and reduce fever. Fexofenadine HCl immediate release 60 mg was chosen because it has a duration of action similar to naproxen sodium (i.e., 12 hours). It is believed that fexofenadine HCl will reduce symptoms of the common cold like runny eyes, runny nose, and sore throat. Fexofenadine may also reduce possible naproxen induced gastrointestinal injury. The latter will be explored in future clinical trials, in addition to the optimal dosing of the drug combination. Together, it is believed that the combination of the two drugs will maximally reduce the virus induced inflammation while having few side effects. The four cases presented here support this belief.

Strengths of these case reports include the fact that one of the cases and her control were married and living together. This implies that several demographic variables of these subjects match. Also, as the cases and controls most likely infected each other, this ensures that both encountered the same type of common cold virus. These factors ensure a more reliable comparison between the cases and controls. Subjects 3 and 4 were their own control and reported on common cold episodes with and without using SJP − 002. An obvious limitation of the case reports is the small sample size, and the fact that the individuals were aware of whether they were taking SJP − 002 or not. Also, common cold status was not verified by any clinical assessment, and thus, the source and type of common cold virus were not determined. For symptom assessment, a modified version of the Jackson Symptoms Scale was used. The item “nasal congestion” replaced the original items “nasal obstruction” and “nasal congestion,” new items included “fever” and “muscle/joint pain,” whereas the item “malaise” was omitted. The scoring of the modified scale (0–10) was also different from the original scale (0 = absent, 1 = mild, 2 = moderate, 3 = severe). Therefore, comparisons with previous research using the Jackson Symptoms Scale are not warranted. For future research, it is recommended to use existing validated scales to assess the severity of symptoms of upper respiratory tract infection. For example, the Wisconsin Upper Respiratory Symptom Survey (WURSS‐44) could be used.^33^ The advantage of this questionnaire is that it also assesses the functional consequences (e.g., performance at work) and impact on quality of life.

Notwithstanding these limitations, the case reports suggest that the use of SJP − 002 was associated with a significant reduction in symptom severity and a shortening of the common cold episode, which warrants further research into the efficacy of SJP − 002.

## AUTHOR CONTRIBUTIONS

**Emina Išerić:** Writing – original draft; writing – review and editing. **Thomas A. Dahl:** Conceptualization; writing – review and editing. **Andrew Scholey:** Conceptualization; writing – review and editing. **Jacqueline M. Iversen:** Conceptualization; writing – review and editing. **Joris C. Verster:** Formal analysis; writing – original draft; writing – review and editing.

## FUNDING INFORMATION

This research received no external funding.

## CONFLICT OF INTEREST STATEMENT

Andrew Scholey has held research grants from Abbott Nutrition, Australian Research Council, Arla Foods, Australian Wine Research Institute, Bayer, Biotechnology and Biological Sciences Research Council, Cognis, Cyvex, European Commission Framework 5, Research and Innovation initiative, GlaxoSmithKline, Ginsana, Kemin Foods, Martek, Masterfoods, National Health and Medical Research Council, Naturex, Nestlé, Neurobrands, Nutricia‐Danone, Red Bull, Sanofi, Verdure Sciences, Wrigley Science Institute, and has acted as a consultant/expert advisor to Abbott Nutrition, Barilla, Bayer Healthcare, Danone, Flordis, GlaxoSmithKline Healthcare, Masterfoods, Martek, Novartis, Sen‐Jam Pharmaceutical, Unilever, and Wrigley. He is Chief Scientific Officer for Arepa Nootropics. Over the past 36 months, J.C.V. has acted as a consultant/expert advisor to Eisai, KNMP, Red Bull, Sen‐Jam Pharmaceutical, and Toast!. T.A.D. is partner and Head of Product Development and Regulatory Affairs of Sen‐Jam Pharmaceutical. J.M.I. is founder and Head of Clinical Development of Sen‐Jam Pharmaceutical. E.I. has no conflicts of interest to declare.

## ETHICS STATEMENT

Not applicable.

## CONSENT STATEMENT

Written informed consent was obtained from the patient to publish this report in accordance with the journal's patient consent policy.

## Data Availability

All available data are presented in this article.
